# Differences in Eotaxin Serum Levels between Polytraumatized Patients with and without Concomitant Traumatic Brain Injury—A Matched Pair Analysis

**DOI:** 10.3390/jcm13144218

**Published:** 2024-07-19

**Authors:** Lukas L. Negrin, Robin Ristl, Gregor Wollner, Stefan Hajdu

**Affiliations:** 1 Department of Orthopedics and Trauma Surgery, Medical University of Vienna, 1090 Vienna, Austria; gregor.wollner@meduniwien.ac.at (G.W.); stefan.hajdu@meduniwien.ac.at (S.H.); 2 Center for Medical Statistics, Informatics and Intelligent Systems, Medical University of Vienna, 1090 Vienna, Austria; robin.ristl@meduniwien.ac.at

**Keywords:** polytrauma, traumatic brain injury, biomarker, eotaxin, CCL11, serum levels, diagnosis

## Abstract

**Background/Objectives**: Early detection of traumatic brain injury (TBI) is crucial for minimizing secondary neurological damage. Our study aimed to assess the potential of IL-4, IL-6, IL-7, IL-8, IL-10, TNF, and eotaxin serum levels—as a single clinical tool or combined into a panel—for diagnosing TBI in multiple injured patients. **Methods:** Out of 110 prospectively enrolled polytrauma victims (median age, 39 years; median ISS, 33; 70.9% male) admitted to our level I trauma center over four years, we matched 41 individuals with concomitant TBI (TBI cohort) to 41 individuals without TBI (non-TBI cohort) based on age, gender, Injury Severity Score (ISS), and mortality. Patients’ protein levels were measured upon admission (day 0) and on days 1, 3, 5, 7, and 10 during routine blood withdrawal using one separation gel tube each time. **Results:** The median serum levels of IL-4, IL-6, IL-7, IL-8, IL-10, and TNF exhibited non-similar time courses in the two cohorts and showed no significant differences on days 0, 1, 3, 5, and 7. However, the median eotaxin levels had similar trend lines in both cohorts, with consistently higher levels in the TBI cohort, reaching significance on days 0, 3, and 5. In both cohorts, the median eotaxin level significantly decreased from day 0 to day 1, then significantly increased until day 10. We also found a significant positive association between day 0 eotaxin serum levels and the presence of TBI, indicating that for every 20 pg/mL increase in eotaxin level, the odds of a prevalent TBI rose by 10.5%. ROC analysis provided a cutoff value of 154 pg/mL for the diagnostic test (sensitivity, 0.707; specificity, 0.683; AUC = 0.718). **Conclusions:** Our findings identified the brain as a significant source, solely of eotaxin release in humans who have suffered a TBI. Nevertheless, the eotaxin serum level assessed upon admission has limited diagnostic value. IL-4, IL-6, IL-7, IL-8, IL-10, and TNF do not indicate TBI in polytraumatized patients.

## 1. Introduction

Traumatic brain injury (TBI) is expected to remain one of the top three causes of injury-related death and disability up to 2030 [1], including cognitive and behavioral changes [2]. In 2019, an estimated 27.16 million people worldwide experienced TBI (95% uncertainty interval (UI), 23.36–31.24 million), and TBI caused 7.08 million (95% UI, ranging from 5.00–9.59 million) years lived with disability [3,4]. The mortality rate for females with severe TBI (Abbreviated Injury Scale (AIS)_Head_ ≥ 3) and concomitant injuries with severity of AIS ≤ 3 was calculated to be 29.2, while for males, it was 30.8 (*p* < 0.002) [5]. Comparing polytrauma patients with an Injury Severity Score (ISS) ≥ 16 and concomitant moderate to severe TBI (AIS_Head_ ≥ 3, AIS of at least one other body region ≥ 3) to patients with isolated moderate to severe TBI (AIS_Head_ ≥ 3, AIS of other body regions ≤ 2) resulted in a higher in-hospital mortality rate in patients with isolated TBI (35% versus 24%, *p* = 0.06), suggesting that fatalities are mainly related to TBI severity regardless of extracranial injuries [6].

A mechanical insult, such as a fall from a great height or a motor vehicle accident, typically causes TBI. Upon impact, contact and inertial forces subject the head to high tensile and shear loads that exceed the mechanical tolerance of the structures. As the brain shifts and rotates inside the bony skull, it causes focal contusions, hematomas, and diffuse axonal injury [7]. This initial physical trauma can provoke several secondary injuries as a result of a chain of neuroinflammatory reactions. This leads to compromised cerebral autoregulation and vasogenic edema by disrupting the blood–brain barrier, ultimately contributing to cerebral ischemia [8].

The impact of TBI is profound and life-changing, not only for survivors but also for their families and caregivers. It places a significant economic burden on the healthcare system, including treatment costs and productivity loss [9]. In polytraumatized patients, who often have a decreased level of consciousness or are under sedation or analgesia, early diagnosis of TBI is crucial to minimize additional secondary neurological injury. Serum biomarker levels are measurable indicators that objectively reflect biological processes and potentially help identify polytraumatized patients with concomitant TBI. During the initial trauma evaluation, blood sampling is a standard procedure. Adding more markers would not require a significant effort. Pilot studies aiming to identify clinically relevant biomarker candidates rely heavily on the trial-and-error principle, leading to positive and negative findings.

The potential of inflammatory cytokines as biomarkers of TBI has been comprehensively studied [10,11,12]. Tumor necrosis factor (TNF) serum [13] and plasma levels [14,15] were significantly higher in TBI patients compared to healthy controls. Significantly elevated interleukin (IL)-4 serum [13] and plasma [16] levels were detected in TBI patients compared to healthy controls. Moreover, IL-4 plasma levels were significantly higher in patients presenting with an ISS > 25 than those with an ISS ≤ 25 [17]. The concentrations of IL-6 in serum [13,18,19] and plasma [15,16] were significantly higher in the TBI group compared to the healthy control group. Additionally, plasma concentrations were significantly increased in multiple injured patients (ISS ≥ 18) than in those with an ISS < 18 at numerous time points [20]. Plasma IL-7 levels were lower in patients suffering from TBI than in healthy controls [16]. IL-8 serum levels were significantly higher [13] and lower [19] in patients with TBI compared to a control group. Individuals suffering from TBI presented significantly higher IL-10 serum [13,19] and plasma [21] levels than healthy controls. Over seven days, trauma patients were monitored for IL-6, IL-8, and IL-10 serum levels. They were divided into three groups (group 1, isolated TBI; group 2, multiple injuries; group 3, multiple injuries including TBI). Group 1 had significantly lower IL-6 serum levels compared to group 2 and group 3. Similarly, IL-8 and IL-10 serum levels were significantly higher in groups 2 and 3 compared to group 1 [22]. Moreover, IL-6 and IL-8 plasma levels were significantly higher in patients suffering from polytrauma and TBI than polytrauma alone [23]. Finally, microdialysis probes were collected from the cortex in a rat model. In animals with inflicted diffuse TBI, a significant increase in ten cytokines, including IL-4, IL-6, IL-10, and TNF, and five chemokines, including eotaxin, was detected compared with sham-injured controls [24].

Chemokines are pivotal in direct chemotaxis [25], migration [26], leukocyte trafficking, inflammatory responses, and immune system functions [27]. The eosinophil chemotactic chemokine eotaxin [28], also known as eotaxin-1 and CCL11 (C-C motif chemokine ligand 11), is a pro-inflammatory cytokine [29], which acts primarily through CCR 3 (C-C motif chemokine receptor 3) [30,31]. Eotaxin was initially believed to selectively mobilize eosinophils locally from the microcirculation or rapidly from the bone marrow [32,33], subsequently recruiting them into inflammatory sites [31,33,34,35,36]. However, there is now evidence that eotaxin may also be chemotactic for basophils [37], Th2 lymphocytes [38], mast cells [39], neutrophils, and macrophages [40]. Eotaxin is present throughout the human body [41]. It is constitutively expressed at high levels in a normal human small bowel and colon and to a lesser extent in the heart, kidney, and pancreas. Detectable levels were observed in the thymus, spleen, liver, lung, prostate, ovary, placenta, and skeletal muscle [36]. In certain inflammatory conditions, eotaxin is released by various types of cells [34], including eosinophils [36,41], endothelial cells [36,42], epithelial cells [42,43], fibroblasts [44,45], keratinocytes [46], and chondrocytes [47,48]. High levels of eotaxin have been described in allergic rhinitis [49], allergic conjunctivitis [50], atopic dermatitis [51], asthma [52], chronic [53] and acute [54] liver diseases, rheumatoid arthritis [55], periodontitis [45], and gastrointestinal disorders [42]. Increased levels of eotaxin have been detected in numerous neuro-inflammatory disorders such as multiple sclerosis [56], as well as neurodegenerative and neuroprogressive disorders including Alzheimer’s disease [57], psychiatric illnesses including significant depression [58], bipolar disorder [59] and schizophrenia [60], and neurocognitive disorders in aging [61]. The available data indicate that serum eotaxin may serve as a potential biomarker for cancer detection [62,63,64,65] and assessing treatment effectiveness [66]. Age was associated with higher plasma levels in patients with mild TBI [14] and higher serum levels in healthy and allergic people [67]. In the last two groups, eotaxin serum levels were significantly higher in males than in females [67]. Contrarily, eotaxin levels did not differ between males and females in fresh frozen plasma and erythrocyte concentrate but significantly increased with the donor’s age in both genders [68].

Eotaxin has rarely been evaluated in a trauma setting. A total of 207 patients with clinically confirmed mild TBI who presented to an emergency department or general practitioner were compared to 82 age-, sex-, and education-matched community controls. Eotaxin plasma levels were obtained at arrival, after two weeks, three months, and 12 months. The levels were significantly higher in patients at all time points compared to the control group [14]. Eotaxin plasma levels were measured in 166 patients with isolated TBI upon admission and at 6, 12, and 24 h post-injury. Median eotaxin levels decreased significantly at 12 and 24 h post-injury only in patients with severe TBI when compared to 21 healthy controls. Additionally, fatalities had significantly higher median eotaxin levels at admission and 6 h post-injury than survivors [21]. In severely injured adult trauma patients, eotaxin serum levels were measured at eight distinct time points within the first 72 h after arrival at the hospital. The median levels were found to be significantly higher in fatalities compared to survivors at all time points [69]. Contrarily, median eotaxin plasma levels assessed upon admission did not show significant differences between 180 polytraumatized patients (median ISS, 26) and 12 healthy controls [70]. Also, the median eotaxin plasma level of 15 clinical TBI patients was similar to those of 19 healthy controls matched in demographics [71]. However, in 16 infants (less than one-year-old) with mild inflicted TBI and 20 infants without brain injury as controls, the median eotaxin serum level was found to be significantly higher in the control group [72]. Finally, the median eotaxin serum level was significantly increased in male mice 24 h after TBI was induced using a mild-to-moderate controlled cortical impact [73]. 

Given the results presented in the literature, we decided to include eotaxin, IL-4, IL-6, IL-7, IL-8, IL-10, and TNF in our search for potential biomarkers to improve the care of multiple trauma victims. The objective of our study was to compare the time courses of the protein serum levels in polytraumatized patients with and without concomitant TBI within ten days of sustaining the injuries and to evaluate whether eotaxin serum levels might be clinically valuable in polytrauma care for TBI diagnosis.

## 2. Materials and Methods

From 1 September 2018 to 31 August 2022, we prospectively enrolled 110 patients using the following criteria for inclusion: a minimum age of 18 years, at least two injuries resulting in an ISS ≥ 16, direct admission to the resuscitation room of our level I trauma center within one hour after the injury occurred, and a stay in the intensive care unit (ICU) for at least one night. Patients with chronic inflammatory lung diseases or malignancies were excluded. In computed tomography scans, TBI was defined as the presence of structural brain damage, such as hemorrhage, swelling, compression, or fractures. We classified each sustained injury using the AIS for the six body regions relevant for calculating the ISS. AIS_Head_ refers to head and neck injuries, including brain and cervical spinal cord, skull fractures, cervical spine fractures, asphyxia, and suffocation. AIS_Face_ classifies injuries to the facial skeleton, nose, mouth, eyes, and ears. AIS_Chest_ rates injuries to the rib cage, thoracic spine, diaphragm, and internal organs of the chest, as well as drowning and inhalation injuries. AIS_Abdomen_ encompasses all lesions affecting abdominal organs and the lumbar spine, whereas AIS_Extremities_ assesses injuries to extremities or pelvic girdles such as sprains, fractures, dislocations, and amputations. Finally, AIS_External_ includes, among others, lacerations, contusions, abrasions, burns, electrical injuries, and explosion-type injuries. Out of the enrolled 110 polytraumatized patients (median age, 39 years; median ISS, 33; 70.9% male), we were able to match 41 individuals with concomitant TBI to 41 individuals without TBI based on age, gender, ISS, and mortality.

Blood was collected from each polytraumatized patient during the initial examination upon admission (day 0) and then on days 1, 3, 5, 7, and 10 during routine blood withdrawal using one separation gel tube (Vacuette R© 8 mL; Greiner Bio-One International; Austria) each time. Immediately after collection, the blood samples were centrifuged at 3000× *g* for 15 min at room temperature to separate the serum, which was then isolated and stored at −80 °C until assayed. We used the 7-Plex Luminex^®^ Performance Human XL Cytokine Panel (Catalog no.: F CSTM18B-07; R&D Systems; Minneapolis, MN, USA) and the Luminex^®^ 200™ Instrument (Catalog no.: LX200-XPON-RUO; R&D Systems; Minneapolis, MN, USA). All measurements were performed in duplicate, and mean values were calculated. The patients were informed about blood sampling as soon as possible. If the patient had a legal guardian, or if one was appointed after the trauma, the guardian was involved in the consent process. In case written consent was not provided, no further blood samples were taken, and the previously sampled material was destroyed upon request from the patient or the guardian.

We utilized IBM SPSS Statistics 29 for statistical analysis and visualization and conducted an a priori analysis [74]. With a power of 0.8 and a significance level of 0.05, we calculated that a minimum sample size of 28 patients per group is required for a two-sample Mann–Whitney U test. Since they are not normally distributed, demographic data and protein serum levels are presented by median and range in square brackets. Qualitative data are described in terms of frequency and percentage. Mann–Whitney U tests were used to compare independent groups, and Wilcoxon signed-rank tests were conducted to compare protein levels within a patient across different time points. The Chi-square test was applied to analyze categorical data. Spearman’s correlation coefficients (ρ) were calculated to reveal associations between protein levels at admission and selected parameters. Moreover, we conducted a univariable binary logistic regression analysis to assess whether the eotaxin day 0 level may indicate TBI in polytraumatized patients. The odds ratio (OR) is presented with a 95% confidence interval (CI). Finally, the receiver operating characteristic (ROC) curve was plotted for graphical analysis, and the corresponding value of the area under the curve (AUC) was determined. The maximum sum of sensitivity and specificity defined the cutoff value. In general, a *p*-value < 0.05 was considered statistically significant.

## 3. Results

The demographic and clinicopathological details of the patients included in our matched pair analysis are presented in Table 1.

### 3.1. Time Course of Median Protein Serum Levels

In the TBI cohort, two patients died on the first day and one each on the fourth, ninth, eleventh, and sixty-second day of hospitalization, respectively. One trauma victim was released on day 10. Consequently, 41, 39, 39, 38, 38, and 36 samples were available for eotaxin level assessment on days 0, 1, 3, 5, 7, and 10. In the non-TBI cohort, one patient died on each of days 1, 4, 42, and 75 during hospital stay. One patient was released on day 8. Therefore, 41, 40, 40, 39, 39, and 38 samples could be assessed on days 0, 1, 3, 5, 7, and 10. 

Table 2 displays the IL-4, IL-6, IL-7, IL-8, IL-10, TNF, and eotaxin serum levels measured at different time points in both the TBI and non-TBI cohorts. Upon admission, only the eotaxin levels differed significantly between the two cohorts.

**Figure 1:**
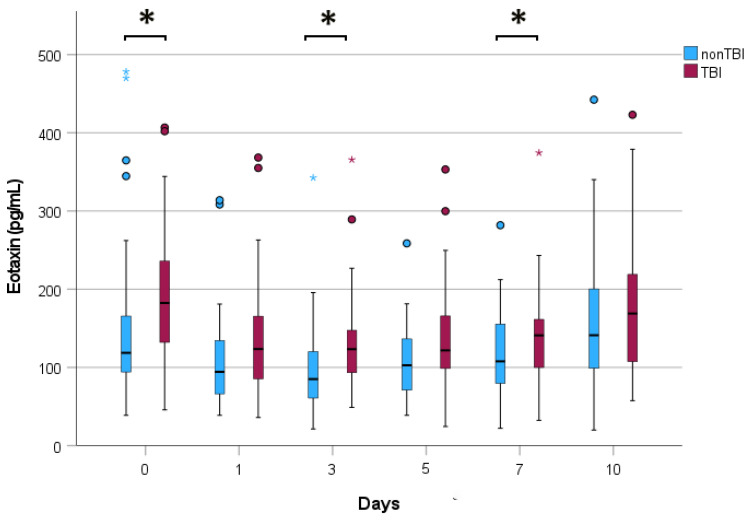
The grouped boxplots display serum eotaxin levels in the TBI and non-TBI cohorts over ten days. The bold horizontal lines within the colored boxes represent the median. The lower and upper limits of the box indicate the 25th and 75th percentiles, defining the interquartile range (IQR). The lower whisker extends to the minor serum level, and the length of the upper whisker is IQR multiplied by 1.5. Circles denote mild outliers, while stars display extreme outliers. A black asterisk indicates a *p*-value of less than 0.05.

Since eotaxin levels were not normally distributed, we visualized the changes in median levels in the TBI and non-TBI cohorts over the study period by grouped boxplots (Figure 1).

### 3.2. Spearman Correlations

Out of all serum levels and parameters assessed, the eotaxin day 0 level solely correlated with age (ρ = 0.339, *p* = 0.002), length of hospital stay (ρ = −0.282, *p* = 0.010), systolic blood pressure (ρ = 0.231, *p* = 0.037), and the TNF day 0 level (r = 0.256; *p* = 0.036). A moderate correlation was calculated between IL-4/IL-6 (ρ = 0.418, *p* < 0.001), IL-4/IL-8 (ρ = 0.484, *p* < 0.001), IL-6/IL-8 (ρ = 0.493, *p* < 0.001), IL-6/IL-10 (ρ = 0.576, *p* < 0.001), and IL-8/IL-10 (ρ = 0.405, *p* < 0.001). All correlation coefficients are presented as Supplementary Material. 

### 3.3. Univariable Logistic Regression Analysis

We performed a univariable binary logistic regression analysis using the eotaxin level measured upon admission as the independent variable and the presence of a TBI as the dependent variable. The analysis resulted in an odds ratio of 1.005, 95% CI (1.0003−1.010), *p* = 0.039, indicating that increasing the eotaxin serum level by 1 pg/mL increases the odds of a suffered TBI by 0.5%. Therefore, for instance, the odds of a TBI increase by 10.5% when the independent variable unit is 20 pg/mL

### 3.4. ROC Statistics

**Figure 2:**
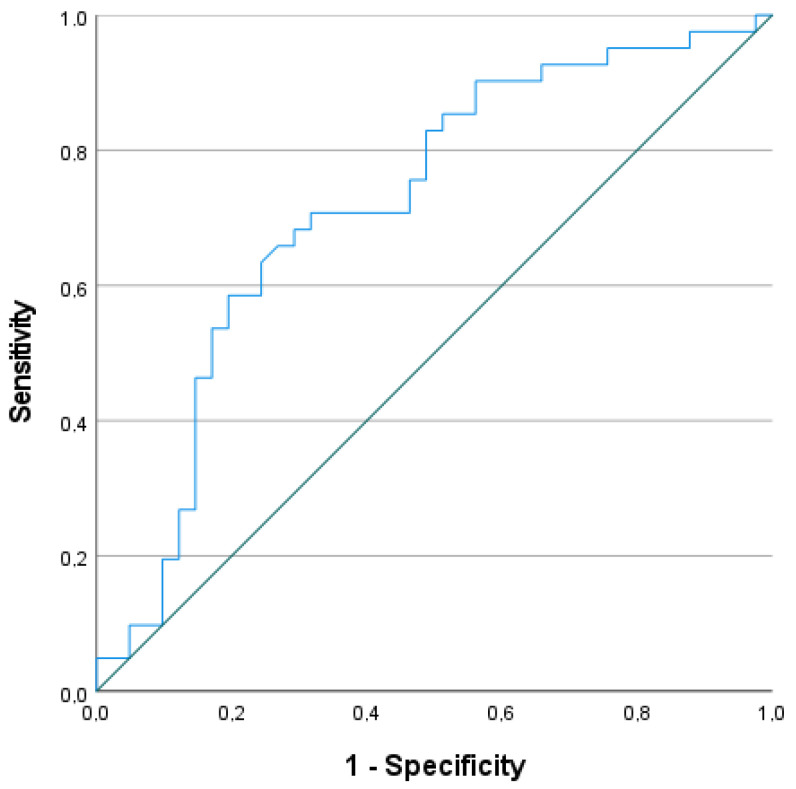
The ROC curve of eotaxin serum level assessed at admission to diagnose TBI in polytraumatized patients.

We plotted the ROC curve for the eotaxin day 0 level and the presence of a TBI for graphical analysis (Figure 2), determining a cutoff value of 154 pg/mL (sensitivity, 0.707; specificity, 0.683) and an AUC of 0.718, 95% CI (0.604−0.831).

## 4. Discussion

Following multiple traumatic injuries, the median eotaxin serum levels exhibited a similar time course in the TBI and the non-TBI cohorts, consistently displaying higher median levels in the TBI cohort throughout the entire observation period. The differences were significant at admission and on days 3 and 5. In both cohorts, the median eotaxin level decreased from day 0 to day 1, then increased until day 10 (*p* < 0.05). Finally, the eotaxin level assessed at admission was significantly associated with the presence of a TBI.

Given that a panel of biomarkers may provide greater sensitivity and specificity for diagnosing TBI compared to a single marker, we evaluated seven promising proteins. Upon admission, we observed significant moderate positive Spearman correlations between several pairs of interleukins, suggesting that the release triggers for these proteins (including mechanisms, sites, extent of injury, and damaged cell types) were somewhat similar. Upon admission, none of the day 0 cytokine serum levels in the TBI cohort showed a significant upregulation and thus cannot be used to indicate TBI in polytraumatized patients.

The eotaxin in the brain of healthy mice is either directly produced by the epithelial layers of the choroid plexus [75] or transported from the blood to the brain tissues; a slow phase of influx precedes the rapid phase without disrupting the blood–brain barrier [76]. In aged mice, eotaxin plasma correlates with reduced neurogenesis, as revealed by tissue processing and immunohistochemistry [77]. In cultured mouse brains, astrocytes [78], pericytes [79], and microglia [80] can be provoked to release eotaxin following exposure to inflammatory mediators. Eotaxin significantly enhances the migration of microglia and can induce the production of reactive oxygen species, which may lead to excitotoxic neuronal cell death and neurological disorders [78]. Therefore, eotaxin may act pathologically by inhibiting endogenous repair mechanisms or augmenting already-established neurodegenerative processes [81].

Up to 30% of individuals who have experienced a mild traumatic brain injury (concussion) continue to experience persistent post-concussion symptoms [82], including headache [83] and depression [84]. In these individuals, who were analyzed two to six months post-trauma, serum eotaxin levels were significantly lower as compared to healthy controls [85]. Following concussion, plasma levels in athletes were significantly lower in females than in males [86]. The concentrations of eotaxin in the dorsolateral frontal cortex were significantly higher in former American football players with chronic traumatic encephalopathy than in non-athlete controls, as assessed in individuals who had donated their brains [87].

Rowland et al. investigated the inflammatory differences between patients with combined polytrauma and TBI and those with only polytrauma. They assessed the plasma levels of various cytokines, including eotaxin, at eight time points during the first three days of admission but did not present results for eotaxin [23]. We were the first to measure eotaxin serum levels in polytraumatized patients with and without TBI within ten days. Table 1 shows no significant differences between the two cohorts in AIS_Face_, AIS_Chest_, AIS_Abdomen_, AIS_Extremities_, and AIS_External_. Therefore, eotaxin levels originating from injuries in these body regions were likely to be similar between the two cohorts, whereas brain injuries seemed to account for the elevated values in the TBI cohort. Unfortunately, we cannot explain the reasons for the decline in eotaxin serum levels from admission to day 1 and, of course, the subsequent rise in both cohorts.

Our discovery of increased eotaxin levels after TBI aligns with findings from one study. Median plasma levels obtained at hospital arrival and after two weeks were significantly higher in patients suffering mild TBI compared to an age-, sex-, and education-matched control group [14]. Contrarily, median eotaxin plasma levels were similar in clinical TBI patients and matched healthy controls [71]. They were even significantly lower in infants with mild inflicted TBI compared to infants with nonspecific symptoms [72]. Furthermore, median eotaxin plasma levels assessed upon admission did not show significant differences between polytraumatized patients and healthy controls [70]. Some studies partly support our findings. Eotaxin plasma levels decreased significantly at 12 and 24 h post-injury in patients with severe TBI compared to the control group [21]. In blunt polytrauma survivors, mean eotaxin serum levels were significantly lower in patients with severe extremity/soft tissue injuries (AIS ≥ 3) four hours, three, four, and seven days after arriving at the hospital compared to those with mild to moderate injuries (AIS < 3) [88]. In patients who had undergone hip replacement surgery, mean eotaxin serum levels were significantly lower compared to their pre-surgery levels during six days following the operation [89], and median eotaxin serum levels were significantly lower 24 h after the arthroplasty [90] compared to their pre-surgery levels.

In a univariable binary logistic regression analysis, a significant association was found between the eotaxin serum level at admission and a TBI in polytraumatized patients. For every 20 pg/mL increase in the eotaxin level, the odds of a present TBI increased by 10.5%. ROC curve analysis yielded a 154 pg/mL cutoff for the eotaxin day 0 level and the presence of a TBI. According to the sensitivity of 0.707 and the specificity of 0.683, the true positive rate using this cutoff level was 70.7%, and the true negative rate was 31.7%. Calculating the AUC is an effective method for summarizing the overall diagnostic accuracy of a test. Unfortunately, 0.718 indicates limited clinical utility for the test [91]. 

Studying serum levels at multiple time points is costly and labor-intensive, with a high risk of yielding negative results. First, pilot studies must identify potential biomarkers. Second, verification and validation using independent sample cohorts are needed, ideally collected in a large multicenter survey to account for clinical and biological variability. This is crucial to determine if there is enough evidence for potential clinical use. Third, kits must be developed to perform tests in the resuscitation room or an affiliated laboratory, providing reliable results within minutes. Our study relates to step 1. It aimed to identify biomarker candidates for TBI in polytrauma patients worthy of further evaluation rather than performing subgroup analyses in the cohorts. According to our a priori analysis, 41 trauma victims in each cohort were sufficient. We did not require expansion since our primary focus was on the significant differences caused by TBI in polytrauma patients. Since our analysis pertained to a trauma setting, we used the non-TBI cohort as the control group for the TBI cohort.

Our study’s limitations include enrolling polytraumatized patients from only one trauma center, insufficient patients to perform subgroup analyses in the matched cohorts, and a lack of an age- and gender-matched control group of healthy individuals.

## 5. Conclusions

Our study’s most important finding is that polytraumatized patients with concomitant TBI have higher eotaxin serum levels compared to those with multiple extracranial injuries, confirming that the brain is a significant source of eotaxin release in humans experiencing a TBI. The time courses from admission to day 10 show a significant decrease followed by a significant increase, warranting investigation in basic research to uncover potential clinical relevance. Despite significant differences in serum eotaxin levels upon admission between the two cohorts and a significant association between the serum eotaxin level on day 0 and a TBI, the chemokine has limited diagnostic value.

## Figures and Tables

**Table 1:** Baseline characteristics of the two cohorts. Displayed values are median [minimum, maximum] or absolute (relative) frequencies.

		TBI	Non-TBI	*p*-Value
Number (n)		41	41	
Age (years)		40 [18, 81]	41 [18, 84]	0.930
Males:females (n)		31:10	31:10	1.000
Fatalities (n)		6 (14.6%)	4 (9.8%)	0.500
ISS		34 [17, 66]	29 [17, 59]	0.357
At admission	Intubated (n)	29 (70.7%)	16 (39.0%)	**0.004**
	GCS of non-intubated	13 [3, 15]	15 [3, 15]	0.072
	Heart rate (BPM)	91 [46, 202]	95 [45, 145]	0.893
	Hemoglobin (g/dL)	11.6 [6.4, 16.2]	10.4 [6.6, 18.1]	0.133
	Lactate (mmol/L)	1.5 [0.4, 27.0]	1.85 [0.7, 12.2]	0.127
	Oxygen saturation (%)	98 [68, 100]	99 [81, 100]	0.856
	pH value	7.33 [6.55, 7.45]	7.33 [6.81, 7.40]	0.647
	Shock index	0.72 [0.36, 6.12]	0.94 [0.43,2.08]	0.144
	Systolic blood pressure (mmHg)	120 [33, 180]	107 [51, 186]	**0.020**
	Blood alcohol concentration (‰)	0 [0, 2.81]	0 [0, 3.36]	0.243
	Abnormal pupils # (n)	22 (53.7%)	8 (19.5%)	**0.001**
	Hypothermia (n)	4 (9.8%)	3 (7.3%)	0.693
AIS_Head_		4 [2, 5]	0 [0, 5]	**<0.001**
AIS_Face_		2 [0, 4]	0 [0, 3]	0.013
AIS_Chest_		3 [0, 5]	3 [0, 5]	0.246
AIS_Abdomen_		0 [0, 4]	2 [0, 5]	0.104
AIS_Extremitis_		2 [0, 4]	3 [0, 5]	0.179
AIS_External_		1 [0, 5]	1 [0, 4]	0.077
Subarachnoid hemorrhage (n)		27 (65.9%)	0 (0%)	**<0.001**
Epidural hematoma (n)		5 (12.2%)	0 (0%)	**0.019**
Intracerebral hemorrhage (n)		16 (30.0%)	0 (0%)	**<0.001**
Subdural hematoma (n)		24 (58.3%)	0 (0%)	**<0.001**
Spine injury (n)		18 (43.9%)	31 (75.6%)	**0.003**
Myelopathy (n)		1 (2.4%)	3 (7.3%)	0.330
Injury causes	Traffic accident (n)	20 (48.8%)	14 (34.1%)	0.109
	Hit by oncoming train/subway (n)	3 (7.3%)	4 (9.8%)	
	Pedestrian hit by vehicle (n)	5 (12.2%)	1 (2.4%)	
	Fall from height (n)	12 (29.3%)	22 (53.7%)	
	Hit by fallen tree branch (n)	1 (2.4%)	0 (0%)	
Suicide attempt (n)		7 (17.1%)	18 (43.9%)	**0.008**
Admitted under resuscitation conditions (n)		3 (7.3%)	1 (2.4%)	0.305
Transport by rescue helicopter (n)		8 (19.5%)	7 (17.1%)	0.775
Under drug influence (n)		6 (14.6%)	5 (12.2%)	0.746
Acute emergency surgery necessary (n)		32 (78.0%)	38 (92.7%)	0.061
Duration of intubation (days)		6 [0, 62]	1 [0, 75]	0.076
Length of stay at the ICU (days)		14 [0, 80]	6 [0, 145]	0.184
Length of stay at the hospital (days)		34 [0, 206]	44 [0, 263]	0.450
Complications (n)		21 (51.2%)	13 (31.7%)	0.073
Sepsis (n)		8 (19.5%)	5 (12.2%)	0.364
ARDS (n)		3 (7.3%)	4 (9.8%)	0.639
Pneumonia (n)		14 (34.1%)	8 (19.5%)	0.135
Acute kidney injury (n)		1 (2.4%)	9 (22.0%)	**0.007**
Hemofiltration (n)		2 (4.9%)	3 (7.3%)	0.664
Urinary tract infiltration (n)		6 (14.6%)	8 (19.5%)	0.557
Pancreatitis (n)		1 (2.4%)	2 (4.9%)	0.556
Clostridium difficile infection (n)		1 (2.4%)	1 (2.4%)	1.000

ISS, injury severity score; GCS, Glasgow coma scale; AIS, abbreviated injury scale; ARDS, acute respiratory distress syndrome. ^#^ Abnormal pupils denote anisocoria and/or absent/abnormal light reactions. Bold indicates *p* < 0.05.

**Table 2:** Serum levels (in pg/mL) of selected proteins. Displayed values are median [minimum, maximum].

		Day 0	Day 1	Day 3	Day 5	Day7	Day 10
IL-4	TBI	18.7 [4.0, 58.0]	18.5 [1.8, 51.4]	14.9 [1.8, 33.9}	14.1 [3.0, 90.9]	13.5 [1.8, 36.3]	10.1 [3.0, 34.0]
	Non-TBI	21.3 [3.9, 98.9]	19.8 [3.5, 60.8]	16.9 [4.7, 49.8]	17.5 [3.3, 46.4]	17.1 [2.2, 43.3]	13.1 [2.2, 48.5]
	*p*-value	0.164	0.490	0.941	0.412	0.600	0.225
IL-6	TBI	59.5 [3.2, 21671,9]	49.1 [5.4, 1539.4]	34.3 [1.3, 1106.2]	14.6 [1.1, 290.7]	12.7 [1.3, 452.3]	9.9 [1.6, 628.7]
	Non-TBI	99.3 [3,1, 766.6]	49.3 [9.8, 564.6]	23.2 [0.6, 967.7]	14.8 [0.5, 664.4]	15.9 [0.8, 213.5]	10.8 [1.1, 262.4]
	*p*-value	0.103	0.639	0.652	0.864	0.717	0.469
IL-7	TBI	7.6 [1.5, 18.2]	7.8 [2.8, 17.8]	7.4 [0.8, 21.5]	9.3 [2.1, 25.6]	10.3 [3.0, 31.1]	15.0 [3.7, 30.2]
	Non-TBI	9.8 [1.7, 19.9]	7.6 [1.3, 19.2]	7.9 [2.2, 17.1]	9.4 [3.8, 25.5]	14.6 [4.7, 40.2]	19.0 [2.6, 36.4]
	*p*-value	0.101	0.625	0.810	0.267	0.071	**0.032**
IL-8	TBI	62.6 [14.6, 15897.0]	42.5 [15.6, 403.5]	34.2 [8.8, 287.6]	37.6 [7.0, 125.3]	31.0 [8.6, 34118.0]	35.3 [12.4, 1763.4]
	Non-TBI	48.7 [11.7, 5374.1]	50.1 [2.8, 816.10]	23.7 [5.7, 311.1]	38.3 [4.4, 474.5]	37.2 [9.6, 404.9]	36.9 [3.8, 1209.4]
	*p*-value	0.985	0.706	0.447	0.800	0.462	0.651
IL-10	TBI	21.6 [0.9, 448.5]	2.5 [0.0, 38.3]	1.4 [0.1, 19.2]	1.1 [0.3, 6.9]	1.2 [0.0, 13.3]	1.4 [0.0, 7.7]
	Non-TBI	34.5 [1.4, 273.8]	3.5 [0.8, 54.2]	1.3 [0.2, 12.1]	1.3 [0.3, 7.7]	1.5 [0.3, 17.5]	1.2 [0.2, 19.8]
	*p*-value	0.250	0.188	0.972	0.732	0.327	0.651
TNF	TBI	4.9 [1.8, 130.9]	6.6 [1.6, 16.1]	5.8 [1.8, 27.6]	6.8 [1.1, 18.1]	4.8 [0.9, 19.2]	5.6 [0.9, 50.2]
	Non-TBI	4.5 [0.1, 8.6]	4.4 [0.3, 11.1]	5.4 [1.6, 16.9]	5.6 [0.4, 14.8]	5.8 [0.4, 15.6]	4.8 [0.6, 17.2]
	*p*-value	0.120	0.079	0.201	0.256	0.897	0.611
Eotaxin	TBI	182.4 [45.8, 2036.3]	121.8 [36.0, 368.4]	123.4 [48.9, 365.7]	121.5 [24.5, 353.2]	141.0 [32.4, 374.5]	172.3 [57.6, 423.1]
	Non-TBI	118.7 [38.8, 478.3]	94.5 [38.8, 313.9]	85.1 [21.3, 342.6]	102.8 [38.8, 258.7]	107.9 [22.2, 281.9]	141.3 [19.9, 442.4]
	*p*-value	**<0.001**	0.098	**0.002**	**0.039**	0.057	0.238

Bold indicates *p* < 0.05.

## Data Availability

The raw data supporting the conclusions of this article will be made available by the authors upon request.

## Supplementary Materials

The following supporting information can be downloaded at https://www.mdpi.com/article/10.3390/jcm13144218/s1, Table S1: Spearman correlation coefficients between protein serum levels and selected clinical parameters.
