# Supplementary material for: Differences in Eotaxin Serum Levels between Polytraumatized Patients with and without Concomitant Traumatic Brain Injury—A Matched Pair Analysis
Source: J Clin Med. 2024 Jul 19;13(14):4218. doi: 10.3390/jcm13144218 (PMC11277900; doi:10.3390/jcm13144218)
Supplement: Supplementary file 1 [file jcm-13-04218-s001.zip › jcm-3078318-supplementary.pdf]

Table S1. Spearman correlation coefficients between protein serum levels and selected clinical parameters.

	Eotaxin d0	IL-4 d0	IL-6 d0	IL-7 d0	IL-8 d0	IL-10 d0	TNF d0	ISS	Heart rate	HB	Lactate	OS	pH	SI	SBP	BAC	DOI	LOS	LOS ICU
Eotaxin d0	1.000	0.135	0.043	-0.103	-0.052	-0.083	0.247*	-0.011	-0.026	0.138	-0.068	-0.050	0.037	-0.166	0.231*	0.100	-0.031	-0.289**	-0.091
IL-4 d0	0.135	1.000	0.418**	0.043	0.484**	0.216	0.118	0.205	0.136	-0.095	0.125	-0.091	-0.098	0.148	-0.184	0.052	-0.078	-0.118	-0.061
IL-6 d0	0.043	0.418**	1.000	0.083	0.493**	0.576**	0.048	0.392**	0.379**	-0.271*	0.300**	-0.233*	-0.298**	0.373**	-0.303**	-0.113	0.166	-0.067	0.116
IL-7 d0	-0.103	0.043	0.083	1.000	-0.026	0.215	-0.086	-0.328**	-0.154	0.158	-0.254*	0.044	0.111	-0.217	0.157	-0.318**	-0.097	0.072	-0.005
IL-8 d0	-0.052	0.484**	0.493**	-0.026	1.000	0.405**	0.147	0.293**	0.164	-0.194	0.332**	-0.099	-0.100	0.309**	-0.396**	0.061	0.085	0.027	0.143
IL-10 d0	-0.083	0.216	0.576**	0.215	0.405**	1.000	0.085	0.306**	0.251*	-0.246*	0.122	-0.071	-0.116	0.278*	-0.242*	-0.244*	0.272*	0.136	0.217
TNF	0.247*	0.118	0.048	-0.086	0.147	0.085	1.000	-0.043	0.054	0.004	0.086	-0.091	-0.135	0.060	-0.053	0.093	0.026	0.168	0.065
ISS	-0.011	0.205	0.392**	-0.328**	0.293**	0.306**	-0.043	1.000	0.344**	-0.371**	0.196	-0.223*	-0.234*	0.356**	-0.286**	0.122	0.359**	0.076	0.321**
Heart rate	-0.026	0.136	0.379**	-0.154	0.164	0.251*	0.054	0.344**	1.000	-0.255*	0.365**	-0.210	-0.265*	0.829**	-0.417**	0.101	0.348**	0.010	0.243*
HB	0.138	-0.095	-0.271*	0.158	-0.194	-0.246*	0.004	-0.371**	-0.255*	1.000	-0.163	0.060	0.267*	-0.396**	0.415**	0.085	-0.161	-0.224*	-0.121
Lactate	-0.068	0.125	0.300**	-0.254*	0.332**	0.122	0.086	0.196	0.365**	-0.163	1.000	-0.164	-0.447**	0.500**	-0.505**	0.466**	-0,128	-0.086	-0.144
OS	-0.050	-0.091	-0.233*	0.044	-0.099	-0.071	-0.091	-0.223*	-0.210	0.060	-0.164	1.000	0.203	-0.142	0.040	-0.048	0.034	0.110	0.002
pH value	0.037	-0.098	-0.298**	0.111	-0.100	-0.116	-0.135	-0.234*	-0.265*	0.267*	-0.447**	0.203	1.000	-0.189	0.134	-0.330**	0.010	0.136	0.114
SI	-0.166	0.148	0.373**	-0.217	0.309**	0.278*	0.060	0.356**	0.829**	-0.396**	0.500**	-0.142	-0.189	1.000	-0.830**	0.183	243*	0.098	0.202
SBP	0.231*	-0.184	-0.303**	0.157	-0.396**	-0.242*	-0.053	-0.286**	-0.417**	0.415**	-0.505**	0.040	0.134	-0.830**	1.000	-0.234*	-0.083	-0.126	-0.090
BAC	0.100	0.052	-0.113	-0.318**	0.061	-0.244*	0.093	0.122	0.101	0.085	0.466**	-0.048	-0.330**	0.183	-0.234*	1.000	-0.061	0.085	-0.078
DOI	-0.031	-0.078	0.166	-0.097	0.085	0.272*	0.026	0.359**	0.348**	-0.161	-0.128	0.034	0.010	0.243*	-0.083	-0.061	1.000	0.468**	0.852**
LOS	-0.289**	-0.118	-0.067	0.072	0.027	0.136	0.168	0.076	0.010	-0.224*	-0.086	0.110	0.136	0.098	-0.126	0.085	0.468**	1.000	0.625**
LOS ICU	-0.091	-0.061	0.116	-0.005	0.143	0.217	0.065	0.321**	0.243*	-0.121	-0.144	0.002	0.114	0.202	-0.090	-0.078	0.852**	0.625**	1.000

d0, day 0; ISS, Injury Severity Score; HB, hemoglobin; OS, oxygen saturation; SI, shock index; SBP, systolic blood pressure; BAC, blood alcohol concentration; DOI, duration of intubation; LOS, length of stay at the hospital; LOS of stay in the ICU.

* The correlation is significant at the 0.05 level (two-sided); ** The correlation is significant at the 0.01 level (two-sided).
